# Overexpression of E2F5/p130, but not E2F5 alone, can inhibit E2F-induced cell cycle entry in transgenic mice

**Published:** 2008-03-25

**Authors:** Qin Chen, Dongcai Liang, Paul A. Overbeek

**Affiliations:** 1 College of Optometry, University of Houston, Houston, TX; 2 Department of Molecular and Cellular Biology, Baylor College of Medicine, Houston, TX

## Abstract

**Purpose:**

The retinoblastoma (Rb) gene family member p130 binds preferentially to the E2F5 transcription factor and forms a repressive E2F5/p130 complex that inhibits cell cycle progression and tumor growth. It is unclear whether E2F5, either alone or in combination with p130, can interfere with the transcriptional activity of other E2F family members, such as E2F1 and E2F3a, in vivo. In this study, we used transgenic mice to test whether overexpression of E2F5 with/without p130 would be sufficient to inhibit E2F1 or E2F3a induced cell cycle reentry.

**Methods:**

Transgenic mice were generated by microinjection of constructs containing different E2F cDNAs (E2F1, E2F3a, and E2F5) or the p130 cDNA linked to the mouse αA-crystallin promoter. The E2F5 single and E2F5/p130 double transgenic mice were cross-mated with E2F1 or E2F3a transgenic mice. The resulting double or triple transgenic mouse embryos were characterized by histology, in situ hybridization, immunohistochemistry, and BrdU incorporation assays.

**Results:**

Overexpression of E2F5 alone was not sufficient to inhibit E2F1 or E2F3a induced cell cycle reentry in lens fiber cells. Transgenic mice coexpressing E2F5 and p130 in lens fiber cells did not show lens defects. However, coexpression of E2F5/p130 with E2F1 or E2F3a in lens fiber cells decreased the number of BrdU positive fiber cells induced by the E2F1 or E2F3a alone. Therefore, overexpression of E2F5/p130, but not E2F5 alone, can inhibit activator E2F-mediated cell proliferation in vivo, confirming that p130 plays a critical role in the repressive activity of E2F5/p130 complex.

**Conclusions:**

Overexpression of E2F5/p130 in post-mitotic lens fiber cells does not affect their normal differentiation program, but can inhibit inappropriate cell cycle reentry induced by the activator E2Fs. Since E2F5 alone cannot interfere with these E2F activities, we conclude that p130 is a key player in the inhibitory process.

## Introduction

The retinoblastoma gene family of tumor suppressors includes three members pRb (Rb1), p107 (RbL1) and p130 (Rb2). Because the main region of sequence similarity between these proteins resides in a pocket domain, they are often referred to as the “pocket proteins” [1]. These proteins play important roles in many aspects of development, particularly in the regulation of cell cycle progression. Although they share many biochemical similarities and have extensive functional overlap, these pocket proteins are not equivalent. For example, in humans, a germ-line mutation of the *Rb1* gene can lead to development of retinoblastoma, a highly malignant intra-ocular tumor that arises in the neural retina of infant eyes. Unlike the *Rb1* gene, the gene encoding p130 is located in chromosome 16q12.2, and deletions in this area are related to several human cancers including prostate, breast, and ovarian cancers [2]. In mice, *Rb1* null embryos die around day 13 of gestation with defects in placental, erythroid, neuronal, and lens development [3,4]. In contrast, mice deficient in p107 or p130 develop normally and exhibit no overt adult phenotypes [5]. The tumor suppressive properties of these pocket proteins are known to be dependent upon their ability to bind to the E2F family of transcription factors and to form a repressive Rb-E2F complex [6-8].

There are currently nine *E2F* genes (*E2F1, E2F2, E2F3a, E2F3b, E2F4, E2F5*, *E2F6, E2F7*, and *E2F8*) identified in mammals [9,10]. Based on their functional considerations, these E2F proteins can be classified into either “activators” (E2F1, E2F2, and E2F3a) or “repressors” (E2F3b, E2F4, E2F5, E2F6, E2F7, and E2F8). The activator E2Fs contain a nuclear localization signal (NLS) and are predominantly in the nucleus. They bind with high affinity to pRb, and their expression is highly upregulated in late G_1_ of the cell cycle. Ectopic expression of any of them is sufficient to induce G_1_/S transition and cell proliferation [9-13]. In contrast, the repressors E2F4 and E2F5 do not have such an NLS domain, therefore, are primarily in the cytoplasm. These two E2Fs bind with high affinity to the pRb homologs, p107 and p130 [8-10]. They are mainly expressed in quiescent cells, and can activate transcription only under some circumstances [9,10,14,15]. The members of E2F6, E2F7, and E2F8, do not have a pocket protein interaction domain and are believed to repress transcription of specific promoters [9,10]. The complexity of the E2F family suggests that individual E2F proteins play distinct roles in the regulation of cell proliferation, as described by our previous observations and many studies from others [9-11,13-15]. However, it is not yet clear whether individual E2Fs could interact with each other. The pRb homolog p130 is thought to be a tumor suppressor, and overexpression of p130 can inhibit cell proliferation by forming a strong repressive complex with E2F4 and E2F5 [2,9,16-20]. Knockout studies showed that E2F4 and E2F5 are necessary for the pocket protein-mediated G_1_ control of the cell cycle, because mouse embryonic fibroblasts (MEFs) deficient for these two E2Fs are unable to exit the cell cycle in response to p16^INK4A^ [21]. However, so far, there is no in vivo evidence showing that extra E2F4 or E2F5, in the absence or presence of extra p130, can interfere with the activities of activator E2Fs. Here, we test whether overexpression of E2F5 with/without p130 is sufficient to repress cell cycle reentry induced by activator E2Fs in post-mitotic lens fiber cells.

**Figure 1:**
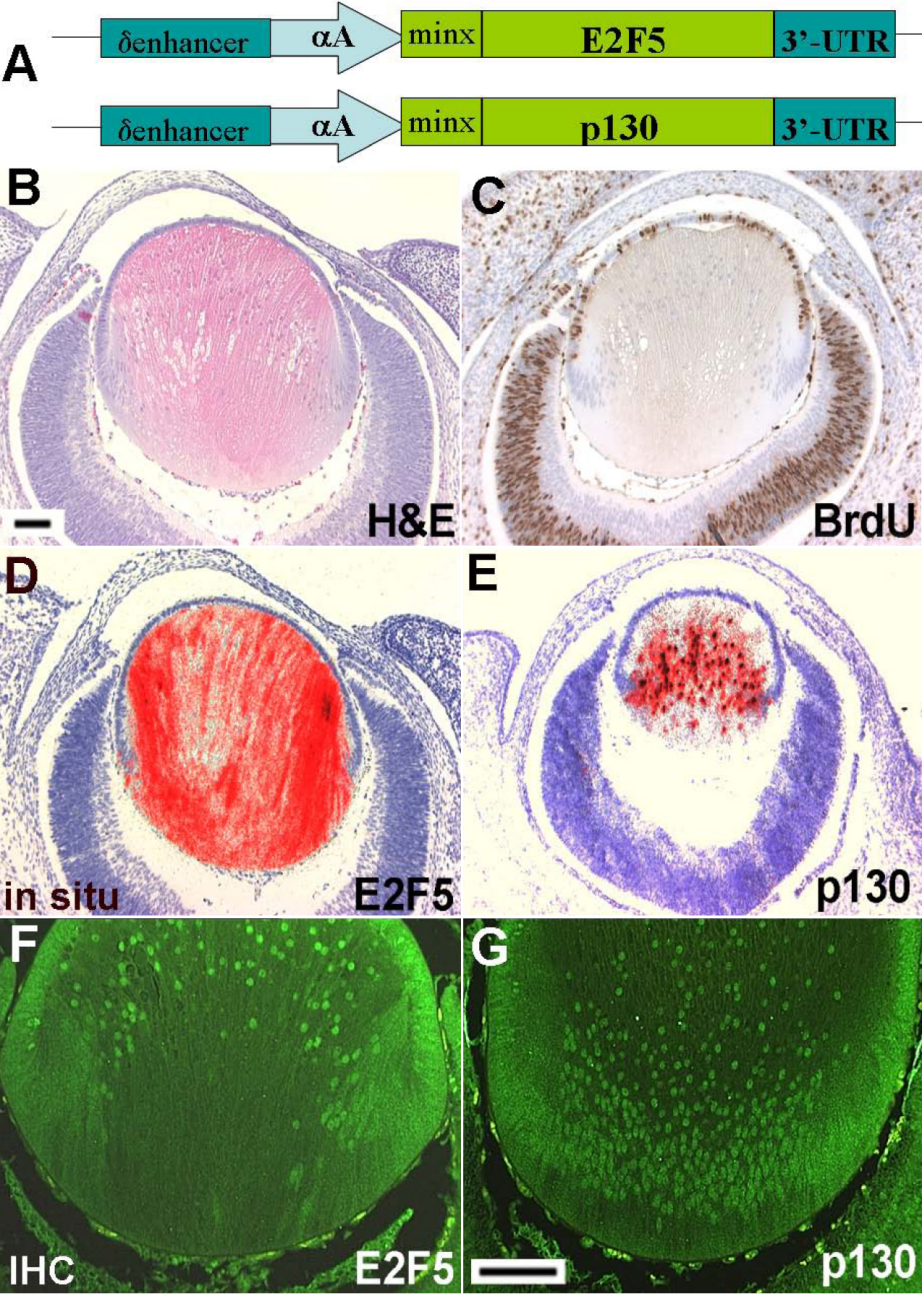
Generation and characterization of E2F5/p130 double transgenic mice. **A:** Microinjected constructs. Human E2F5 and mouse p130 cDNAs were linked to the δenhancer αA-crystallin promoter in vector δenαA-minx (DREAM) [30]. **B:** At E15.5, the bigenic lens shows a normal lens phenotype. **C:** No BrdU positive fiber cells were present in the center of the double transgenic lens. **D-E:** In situ hybridization was used to examine expression of E2F5 and p130 transgenes with human E2F5 and mouse p130 riboprobes. Hybridization signals were initially captured as dark-field images, pseudocolored red, and then superimposed on bright-field images of the same tissue sections counterstained by hematoxylin. The E2F5 (**D**) and p130 (**E**) transgenes were expressed in lens fiber cells. The size of lens in panel E is smaller as this section is more peripheral. **F-G:** Immunohistochemistry. Using antibodies against E2F5 (**F**) and p130 (**G**), a green nuclear staining was detected in the double transgenic lens fiber cells. Scale bars=500 μm.

**Figure 2:**
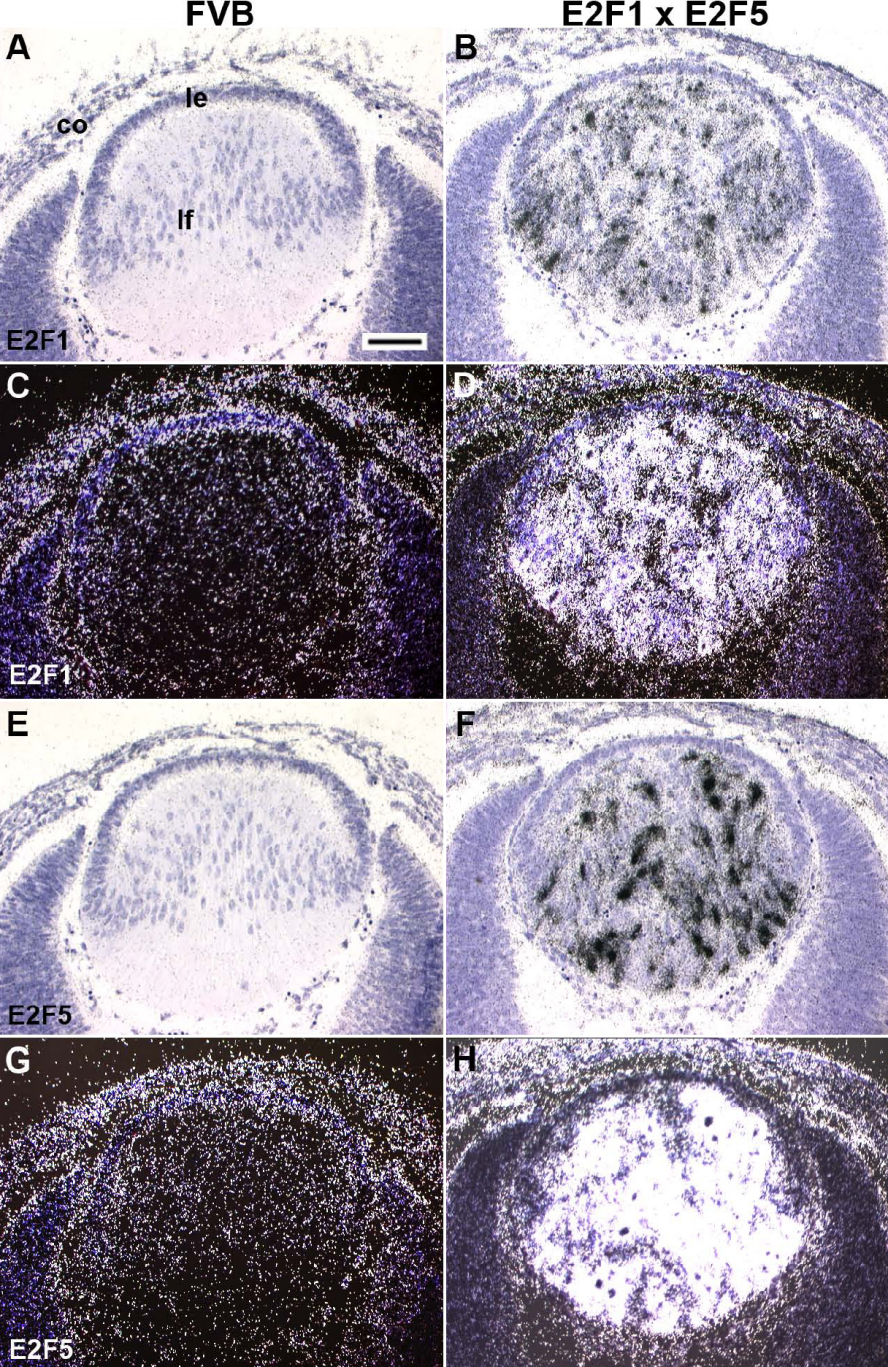
Expression of transgenes in E2F1/E2F5 bigenic eyes at E14.5. In situ hybridizations with human E2F1 and E2F5 riboprobes were used to confirm expression of E2F1 and E2F5 transgenes. Hybridization signals were captured as bright- (**A**, **B**, **E**, **F**) and dark- (**C**, **D**, **G**, **H**) field images. Transcripts of the endogenous E2F1 and E2F5 were detected in non-transgenic FVB lens epithelial and corneal cells (**C**, **G**). Endogenous E2F5, but not E2F1, was also detectable in the FVB lens fiber cells (**C**, **G**). By comparison, both transgenes were highly expressed in the bigenic lens fiber cells (**B**, **D**, **F**, **H**), as described previously [15,29]. Scale bars=500 μm.

The embryonic lens of the eye is an attractive model system for studying the molecular mechanisms that regulate cell proliferation and differentiation. The lens is composed of two cell types: anterior proliferative epithelial cells and posterior terminally differentiated, post-mitotic, elongated fiber cells [22]. Lens epithelial cells at the bow region are stimulated to exit from cell cycle and to differentiate into fiber cells. Almost all E2F family members are expressed in lens epithelial cells, while only E2F1, E2F3 and E2F5 are detected in post-mitotic lens fibers [23]. pRb and p107 proteins were detected in both lens epithelial and fiber cells [23]. Since p130/E2F complexes were not detected in lens fiber cells, it was proposed that pRb and p107 are the primary regulators of E2F activities in differentiating lens fiber cells [23]. Previous studies have shown that inactivation of pRb by targeted mutagenesis of the *Rb* gene, or by expression of viral proteins, results in inappropriate cell cycle reentry in lens fiber cells [24-27]. The activator E2Fs, particularly E2F3, make a major contribution toward the in vivo phenotypic consequences of pRb deficiency [28]. Although both E2F5 and p130 are considered to play important roles in the regulation of cell cycle exit and terminal differentiation, it is not known whether elevated levels of E2F5 in combination with p130 would be able to alter lens fiber cell differentiation program.

In this study, we have generated double transgenic mice with lens-specific coexpression of E2F5 with E2F1 or E2F3a. We have also generated transgenic mice coexpressing E2F5 with p130 in the lens fiber cells, and triple transgenic mice coexpressing E2F5/p130 with E2F1 or E2F3a. We found that overexpression of E2F5/p130 in the lens fiber cells did not alter normal fiber cell differentiation. Overexpression of E2F5 alone was not sufficient to repress inappropriate cell cycle entry induced by E2F1 or E2F3a. However, cell cycle reentry was significantly inhibited by overexpression of E2F5/p130.

**Figure 3:**
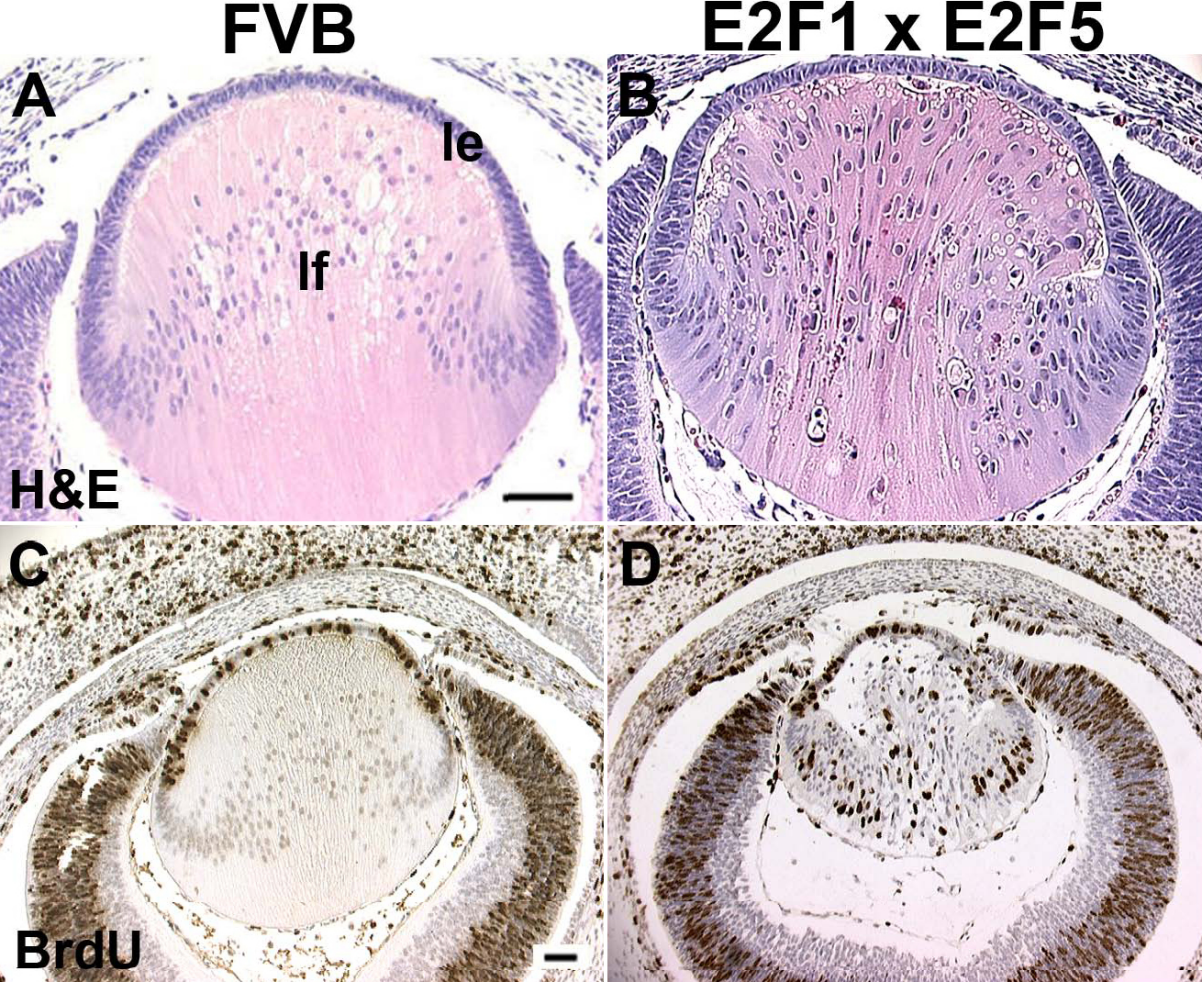
Characterization of E2F1/E2F5 bigenic eyes at E14.5. When compared with FVB lens (**A**), the bigenic lens histology shows alterations in fiber cell elongation, and the presence of extra nuclei in the center of the lens (**B**), similar to the lens phenotype in the E2F1 single transgenic lens [29]. There are no BrdU positive fiber cells (brown nuclear stain) in the FVB lens (**C**). BrdU positive fiber cells were prevalent in the double transgenic lens (**D**). Abbreviations: le, lens epithelium; lf, lens fiber. Scale bars=500 μm.

## Methods

### Generation of the constructs and transgenic mice

All animals were used in accordance with the ARVO Statement for the Use of Animals in Ophthalmic and Vision Research. Human E2F1, Myc-tagged mouse E2F3a and human E2F5 transgenic lines were generated as previously described [15,29]. E2F5/p130 double transgenic mice were generated by coinjection. A Sac II – Xba I fragment containing the human E2F5 cDNA and a Cla I – EcoR V fragment carrying mouse p130 cDNA were cloned downstream from the δenhancer/αA-crystallin promoter in the vector δenαA-minx (DREAM) [30]. The resultant plasmids (Figure 1A) were digested with *Kpn*I and NotI to release fragments for microinjection. The fragments were separated by electrophoresis through a 1.2% agarose gel and purified using a Qiaex II gel extraction kit (Qiagen, Hilden, Germany). Transgenic mice were generated by pronuclear coinjection of both fragments into one-cell-stage inbred FVB/N embryos [31,32]. Two stable transgenic families (OVE1811 and OVE1812) expressing both E2F5 and p130 transgenes were generated from the coinjection. E2F5 single (OVE1598) and E2F5/p130 double (OVE1811) transgenic mice were cross-mated with E2F1 (OVE527) or E2F3a (OVE1728) transgenic mice to generate double (E2F5/E2F1 or E2F5/E2F3a) and triple (E2F5/p130/E2F1 or E2F5/p130/E2F3a) transgenic mice.

### Screening of transgenic mice

Genomic DNA from mouse tails was isolated as previously described [31]. For polymerase chain reaction (PCR) screening of E2F1, E2F3a, and E2F5 single transgenic mice, sense (5′-GTG AAG GAA CCT TAC TTC TGT GGT G) and antisense (5′-GTC CTT GGG GTC TTC TAC CCT TTC TC) primers specific to the simian virus (SV) 40 sequences in CPV2 were used to amplify a ~300 bp fragment. To screen for the E2F5 and p130 transgenes driven by the δenhancer/αA-crystallin (DREAM) promoter, we used the following primers: 1) minx sense: 5′-GTC TTT CCA GTG GGG ATG CTC T-3′; 2) E2F5 antisense: 5′-TCC TGC AGC AGC GAC ACG AA-3′; 3) p130 antisense: 5′-GCT GCT GGA TCT GAT GGC TC-3′. A ~400 bp fragment was amplified by the minx/E2F5 or minx/p130 primers. PCR assays were performed as described previously [29].

### Lens histology

Female transgenic mice were superovulated and mated with male transgenic mice. The presence of a vaginal plug was defined as gestational day 0.5. Embryonic heads at embryonic day (E) 14.5 and 15.5 were fixed in 10% formalin, paraffin embedded, cut into 5-μm thick sections, and stained with hematoxylin and eosin by standard techniques.

**Table 1:** Percentage of BrdU positive lens fiber cells.

**Transgenic families**	**BrdU^#^ (%)**
E2F1	42±1.6
E2F3a	10±1.2
E2F5	0
E2F1/E2F5	44±1.8
E2F3a/E2F5	10±1.3
E2F5/p130	0
E2F5/p130/E2F1	8±0.8
E2F5/p130/E2F3a	5±0.5

The sharp (hash mark) shows the percentage of positive cells: the number of brown nuclei in lens fiber cells compared with the total number of fiber cell nuclei.

**Figure 4:**
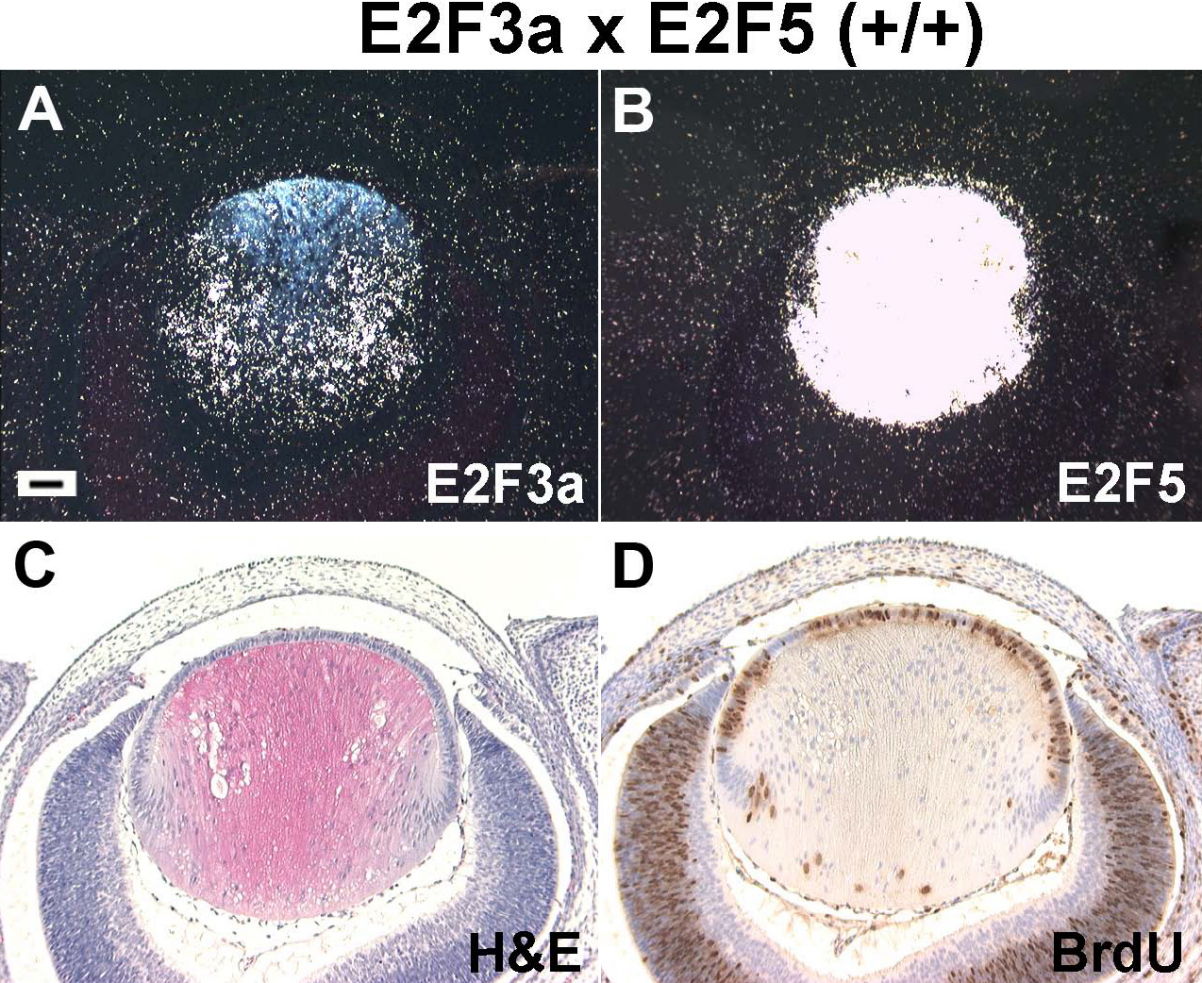
Characterization of E2F3a/E2F5 double transgenic eyes at E15.5. **A, B:** Expression of E2F3a and E2F5 transgenes was confirmed by in situ hybridizations with E2F3a and E2F5 riboprobes. Hybridization signals were captured as dark-field images. The E2F3a (**A**) and E2F5 (**B**) transgenes were specifically expressed in lens fiber cells, as described previously [15]. **C:** Ocular histology of the E2F3a/E2F5 double transgenic lenses showed modest alterations in fiber cell elongation and the presence of extra nuclei in the center of the lens. **D:** The same percentage of fiber cells were BrdU positive in the double transgenic lens as in the E2F3a single transgenic lens described previously (Table 1) [15]. Scale bars=500 μm.

**Figure 5:**
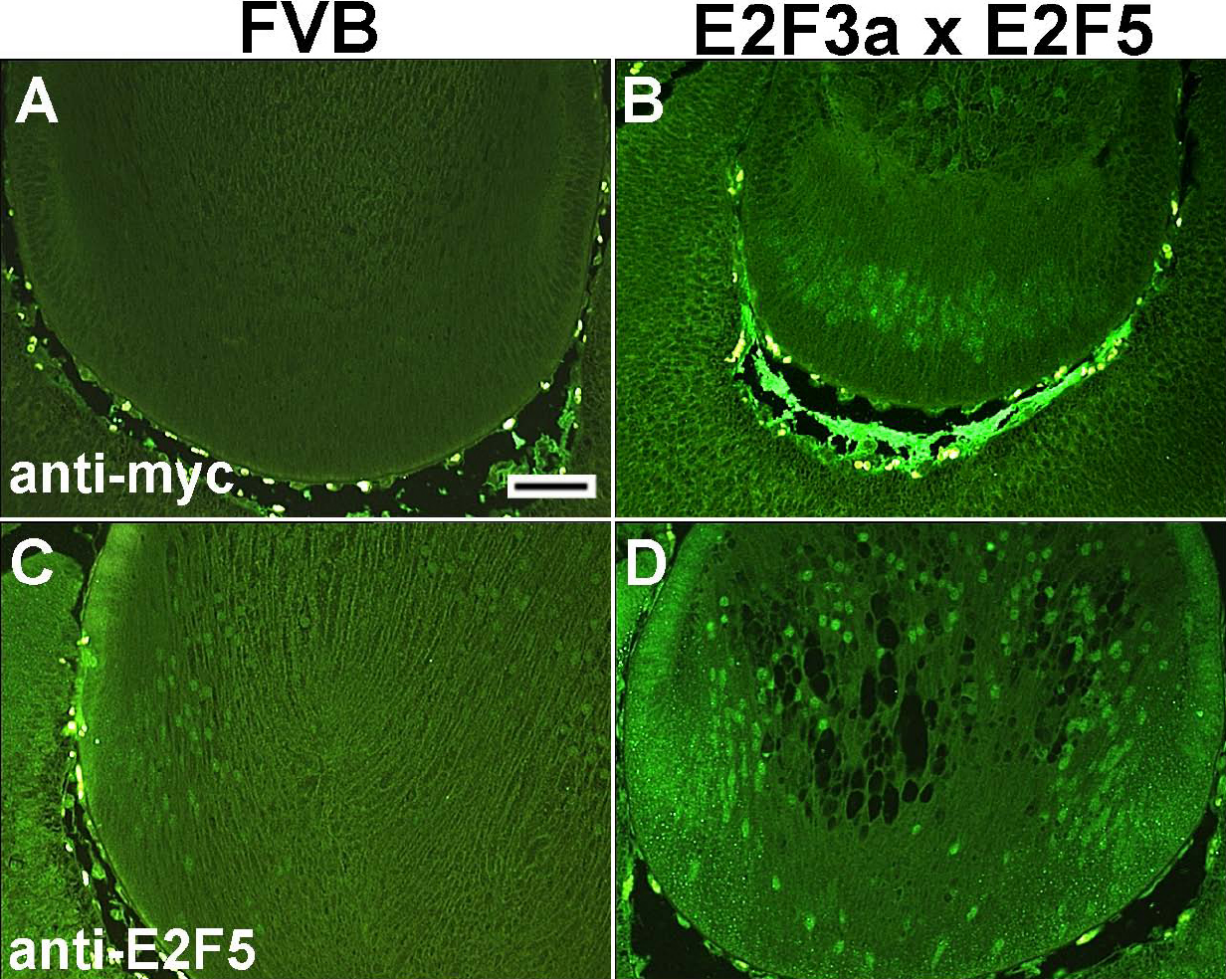
Expression of Myc-E2F3a and E2F5 proteins. Immunohistochemistry was used to assay expression of the E2F3a and E2F5 transgenic proteins in non-transgenic FVB (**A**, **C**) and the E2F3a/E2F5 double transgenic lenses (**B**, **D**). Using anti-Myc antibody, there is no green staining in FVB lens (**A**). By comparison, green nuclear staining is present in the transgenic lens fiber cells (**B**) indicative of Myc-tagged E2F3a expression. When antibody against E2F5 was used, endogenous E2F5 in FVB lens fiber cells was detected as a weak green nuclear staining (**C**). A stronger signal was present in the double transgenic fiber cell nuclei (**D**). Scale bars=500 μm.

### In situ hybridization

In situ hybridization was performed using ^35^S-labeled riboprobes, as described in Fromm and Overbeek [33]. An SV40-specific riboprobe and the probes for E2F1, E2F3a, and E2F5 were described previously [15,29]. A riboprobe for mouse p130 was generated from plasmid p130/pBluscript. Hybridization signals were initially captured as dark-field images. For some of the figures, the dark-field images were pseudo-colored red, then superimposed on bright-field images of the same tissue section (counterstained by hematoxylin) using image analysis software (Photoshop; Adobe, San Diego, CA).

### BrdU Incorporation

DNA replication was detected by 5-bromo-2’-deoxyuridine (BrdU) incorporation. BrdU (100 μg/g bodyweight; Sigma-Aldrich Co., St. Louis, MO) was injected into pregnant female mice. One hour later, the mice were sacrificed and embryos were analyzed by immunohistochemistry as described previously [25]. For quantification, the tissue slides containing mid-frontal lens sections were selected for BrdU staining, and the number of BrdU-positive nuclei in lens fiber cells was counted and compared with the total number of nuclei in the same region, determined by hemotoxylin staining. Results are the mean ± SD from four individual tissue slides (each slide was from different transgenic tissue block and contained at least 3 eye sections). Statistical analyses were performed with two-tailed Student's *t*-tests using Microsoft Excel software. Data were considered to be significantly different for p<0.05.

**Figure 6:**
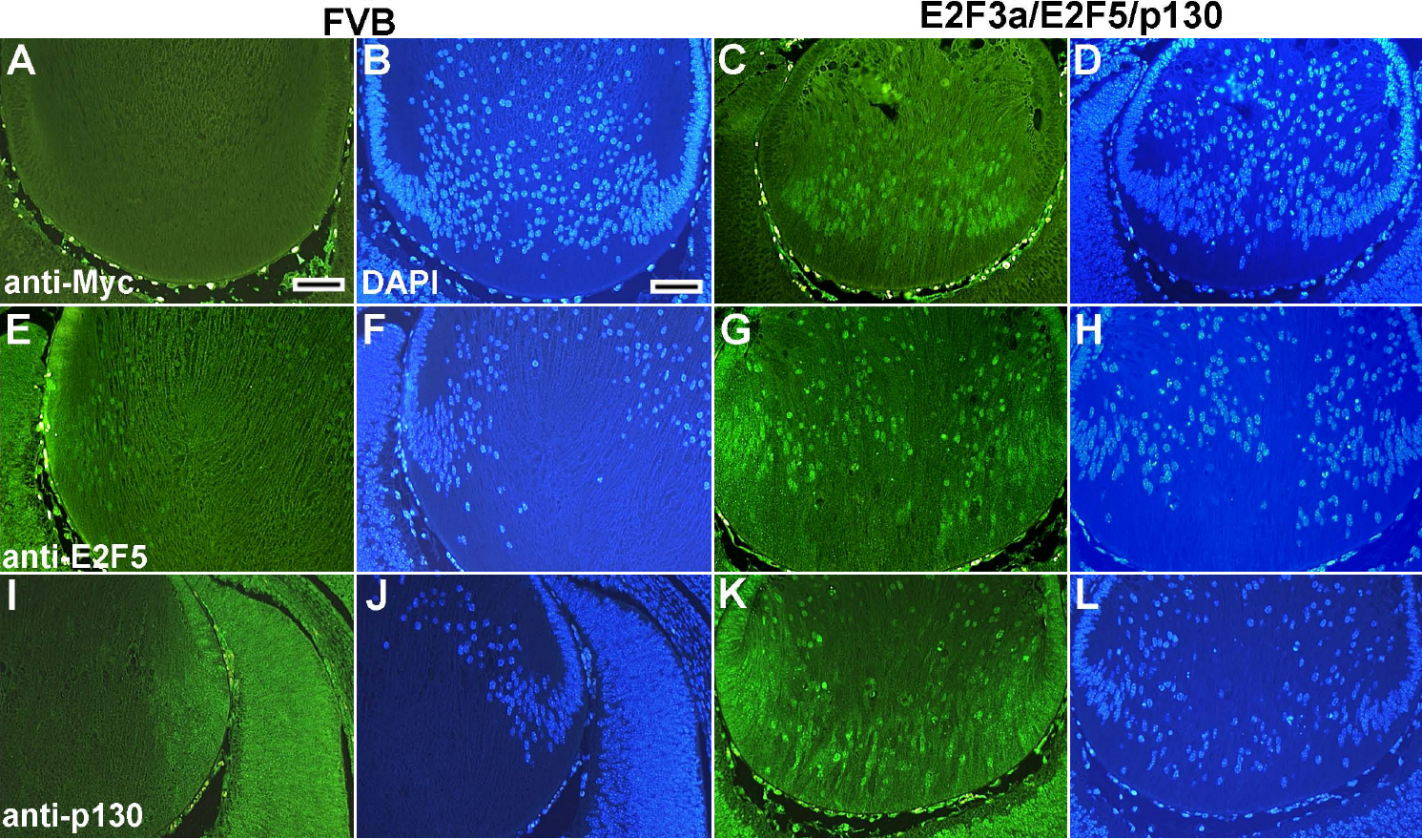
Expression of E2F3a/E2F5/p130 proteins at E15.5. Immunohistochemistry was used to assess expression of E2F3a, E2F5 and p130 proteins in non-transgenic FVB (**A**, **B**, **E**, **F**, **I**, **J**) and triple transgenic lenses (**C**, **D**, **G**, **H**, **K**, **L**). There was no positive staining for Myc-E2F3a (**A**) or p130 (**I**) in FVB lens fiber cells. By comparison, intensive green nuclear staining was seen in the triple transgenic lens fiber cells for all three proteins (**C**, **G**, **K**). Cell nuclei were visualized by DAPI staining (**B**, **D**, **F**, **H**, **J**, **L**). Scale bars=500 μm.

**Figure 7:**
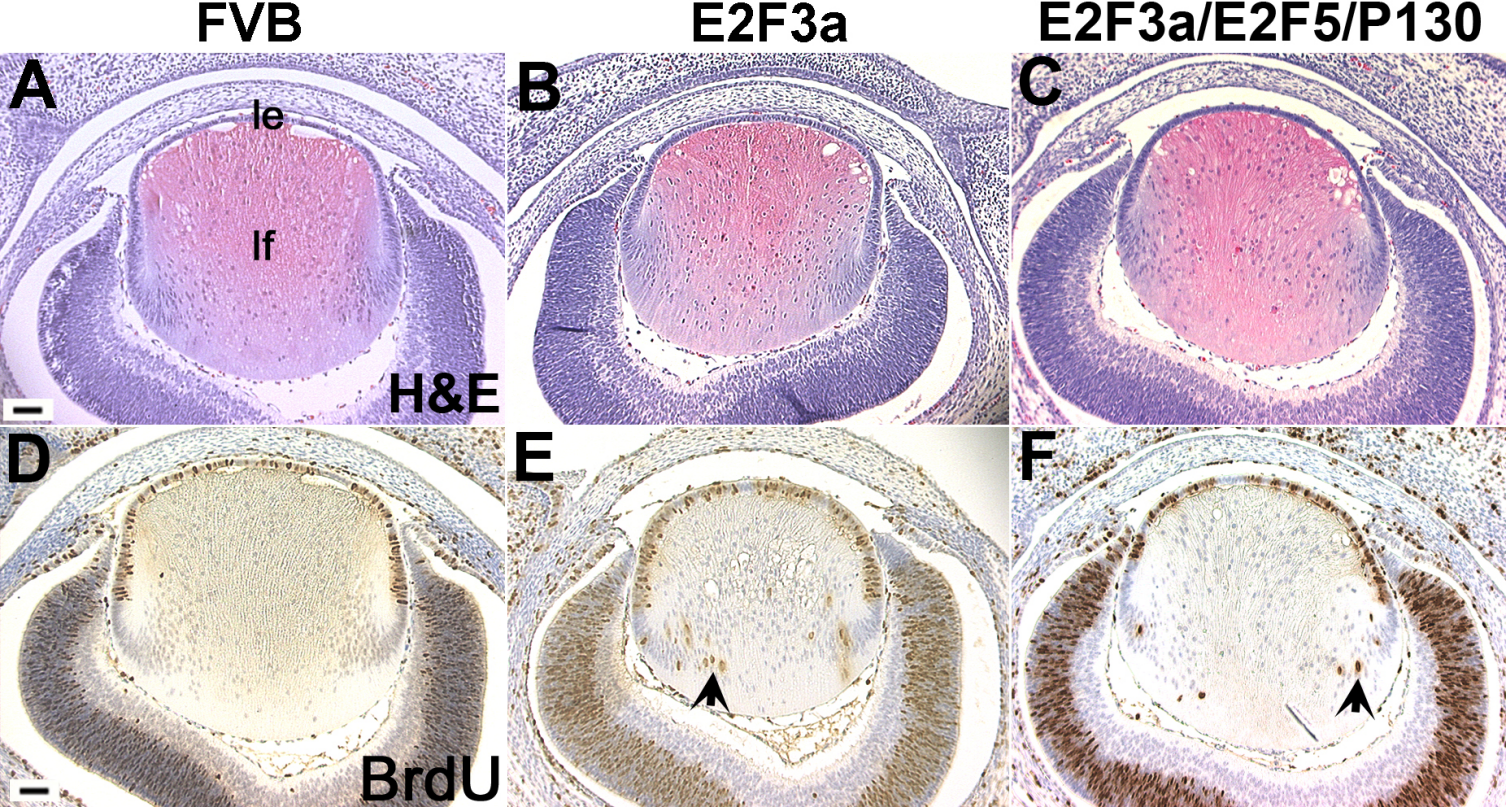
E2F3a/E2F5/p130 triple transgenic lens histology and BrdU incorporation assays. **A-C:** At E15.5, when compared with a non-transgenic FVB lens (**A**), the triple transgenic lens showed a modest phenotype with some displaced fiber cell nuclei(**C**) in contrast to the many fiber cell nuclei seen in the E2F3a single transgenic lens (**B**). Abbreviations: le, lens epithelium; lf, lens fiber. **D-F:** The number of BrdU positive fiber cells (arrow head) in the triple transgenic lens (**F**) decreased by about 50% when compared to the E2F3a single transgenic lens (**E**). Scale bars=500 μm.

**Figure 8:**
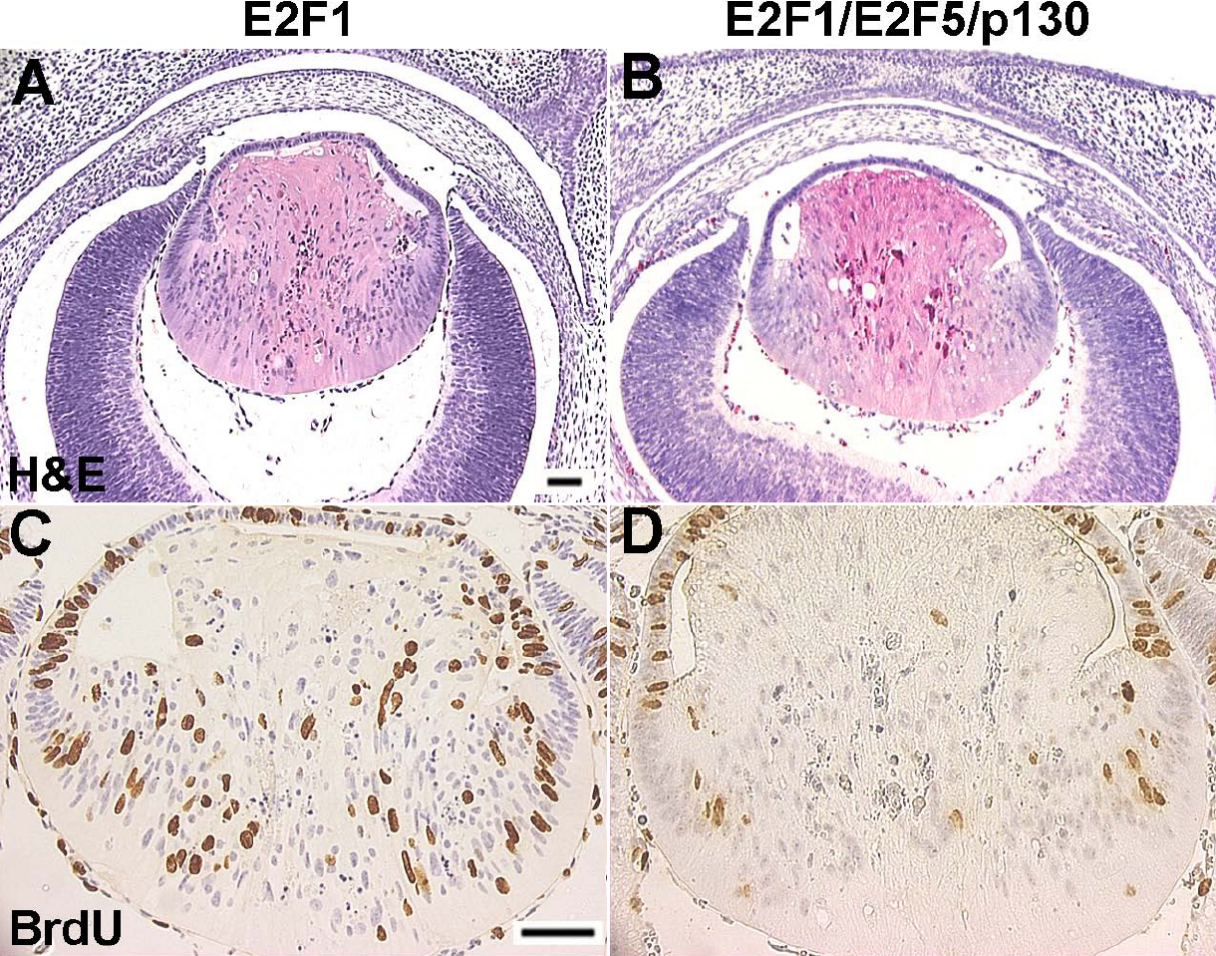
E2F1/E2F5/p130 triple transgenic lens histology and BrdU incorporation assays. **A-B:** At E15.5, the E2F1 single (**A**) and E2F1/E2F5/p130 triple transgenic lenses (**B**) showed similar defects in fiber cell elongation, plus the presence of extra condensed nuclei in the center of the lenses. **C-D:** However, the number of BrdU positive fiber cells in the triple transgenic lens (**D**) had decreased by 80% when compared to the E2F1 single transgenic lens (**C**). Scale bars=500 μm.

**Figure 9:**
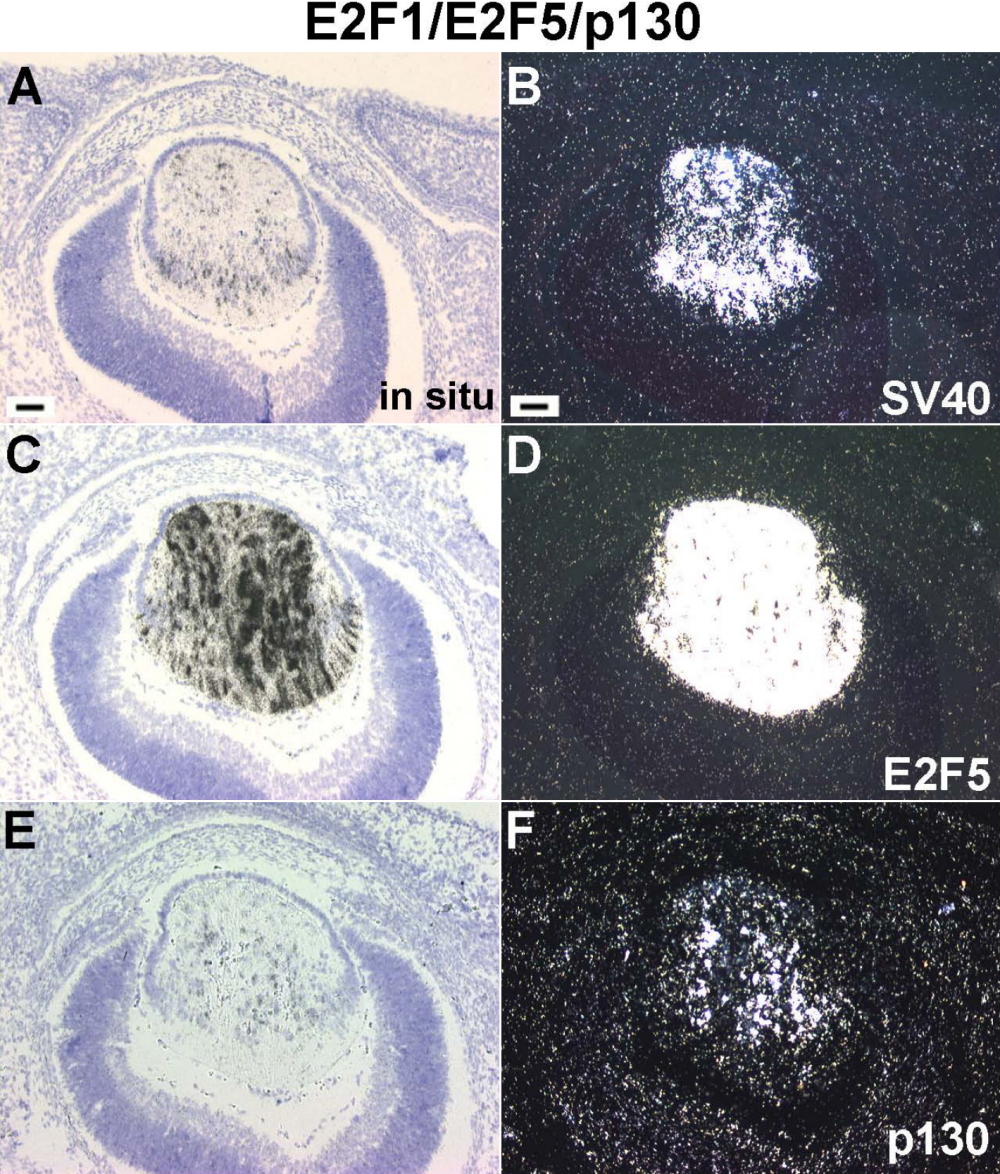
Transcription of E2F1/E2F5/p130 transgenes at E15.5. In situ hybridizations were used to assay expression of the E2F1, E2F5 and p130 transgenes using SV40 (for E2F1), human E2F5 and mouse p130 riboprobes. Bright-field (**A**, **C**, **E**) and dark-field (**B**, **D**, **F**) images are shown. The transgenes were expressed specifically in lens fiber cells. Transcription signals of p130 (**F**) were much weaker than the E2F1 (**B**) and E2F5 (**D**) transgenes. Scale bars=500 μm.

### Immunohistochemistry

Tissue sections were deparaffinized in xylene and rehydrated through a graded alcohol series, washed with PBS, and incubated with 10% methanol/3% hydrogen peroxide in PBS to block endogenous peroxidase activity. Slides were boiled in 10 mM sodium citrate buffer (pH 6.0) for 20 min using a microwave oven, and then incubated with 10% normal horse serum for 1 h at 37 °C. For detection of E2F5, p130, and Myc-tagged E2F3a transgenic proteins, primary antibodies against E2F5 (Santa Cruz Biotechnology, Santa Cruz, CA), p130 (Sigma-Aldrich Co.), or Myc (Sigma-Aldrich Co.) were used at a 1:500 dilution in 10% normal horse serum in PBS, with incubation at 4 °C for 24 h. The slides were washed with PBS (4 × 5 min), and a secondary antibody (1:200; biotin-conjugated anti-rabbit or anti-mouse IgG; Vector Laboratories, Burlingame, CA) was added for 1 h at room temperature. After washing 4 times with PBS, ExtrAvidin-fluorescein isothiocyanate conjugate (Sigma-Aldrich Co.) was added and incubated for 1 h at room temperature, followed by PBS washes. The slides were mounted with Aqua-Poly/Mount (Vector Laboratories).

## Results

### Overexpression of E2F5 does not affect E2F1-induced cell cycle reentry

In our previous studies, we generated transgenic mice specifically expressing E2F1 or E2F5 in lens fiber cells [15,29]. At E15.5, the E2F1 lenses (OVE527 and OVE530) showed severe defects in fiber cell elongation and alignment. At the posterior of these transgenic lenses, there were many extra nuclei which incorporated BrdU indicative of inappropriate cell cycle reentry, and many condensed fiber nuclei indicative of apoptosis [29]. By comparison, the transgenic mice in each E2F5 family (OVE1598 and OVE1599) showed a normal eye phenotype, and there were no extra BrdU positive nuclei in the E2F5 expressing lens fiber cells [15]. Since E2F5 is thought to be a cell cycle “repressor,” we tested whether overexpression of E2F5 was sufficient to inhibit the E2F1-induced cell cycle reentry. Homozygous E2F1 mice (OVE527) were cross-mated with E2F5 transgenic mice (OVE1598) to generate E2F1/E2F5 double transgenic mice. To confirm expression of transgenes, in situ hybridization was performed using riboprobes for human E2F1 or E2F5. At E14.5, transcripts of the endogenous E2F1 and E2F5 were detected in non-transgenic FVB lens epithelial and corneal cells (Figure 2C,G), suggesting that these human E2F1 and E2F5 riboprobes cross-reacted to the mouse E2F1 and E2F5 transcripts. Endogenous E2F5, but not E2F1, was also detectable in the FVB lens fiber cells (Figure 2C,G). By comparison, E2F1 and E2F5 transgenes were highly expressed in the double transgenic lens fiber cells (Figure 2B,D,F,H). The double transgenic lenses showed defects in fiber cell elongation (Figure 2B), and many BrdU positive lens fiber cells (Figure 3D). The percentage of BrdU positive fiber cells between E2F1 single and E2F1/E2F5 double transgenic lenses was not significant (p>0.05; Table 1). Therefore, overexpression of E2F5 alone is not sufficient to repress E2F1-induced cell cycle reentry.

### Overexpression of E2F5 does not affect E2F3a-induced cell cycle reentry

E2F3a transgenic mice have small eyes and cataracts, similar to the E2F1 transgenic mice with BrdU positive fiber nuclei and enhanced programmed cell death [15,29]. To determine whether overexpression of E2F5 could alter the E2F3a phenotype, E2F3a transgenic mice (OVE1728) were cross-mated with the E2F5 transgenic mice (OVE1598) to generate the E2F3a/E2F5 double transgenic mice. At E15.5, in situ hybridization revealed that both transgenes were expressed in lens fiber cells, and that E2F5 was more highly expressed at the RNA level (Figure 4A,B). Lens morphology and the number of BrdU positive fiber cells in the E2F3a/E2F5 double transgenic lenses were similar to that seen in the E2F3a single transgenic lenses (p>0.05; Figure 4C,D; Table 1) [15]. To verify expression of both transgenic proteins, immunohistochemistry was performed using anti-Myc antibody for the E2F3a and anti- E2F5 for the E2F5 transgene. As shown in Figure 5, there was no staining for Myc-tagged protein in the non-transgenic lens (Figure 5A). By comparison, immunofluorescent staining was seen in the nuclei of the transgenic lens fibers confirming expression of the E2F3a transgene (Figure 5B). We previously showed that in the non-transgenic FVB lens, there was weak staining for endogenous E2F5 protein in fiber cell nuclei (Figure 5C) [15]. The intensity of E2F5 staining was stronger in the transgenic lens (Figure 5D), indicating the presence of extra transgenic E2F5 protein. Therefore, overexpression of E2F5 alone is not sufficient to inhibit E2F3a-induced cell cycle reentry.

### Generation of E2F5/p130 double transgenic mice

The pRb homolog p130 is a binding partner of E2F5. It has been known that overexpression of p130 can lead to a G_1_ cell cycle arrest in cultured cells [34]. Since E2F5 and p130 complex are thought to be important in the regulation of cell cycle exit and terminal differentiation, we wanted to test whether coexpression of E2F5 and p130 would be able to alter normal lens fiber cell differentiation. To generate transgenic mice expressing both E2F5 and p130, we co-injected DREAM-E2F5 and DREAM-p130 constructs (Figure 1A). Two stable bigenic families (OVE1811 and OVE1812) were obtained from the coinjection. Mice in both families showed a normal eye phenotype.

### Overexpression of E2F5/p130 does not affect normal lens fiber cell differentiation

The lens morphology in the E2F5/p130 double transgenic embryos at E15.5 was similar to that seen in nontransgenic or E2F5 only transgenic lenses (Figure 1B) [15]. There was no BrdU incorporation detected in the bigenic lens fiber cells (Figure 1C). In situ hybridization revealed that transcripts of E2F5 and p130 were present specifically in the transgenic lens fiber cells (Figure 1D,E). A previous study showed that E2F/p130 complexes were undetectable in rat lens fiber cells, probably due to degradation of the p130 protein by ubiquitination [23]. To test the presence of p130 and E2F5 transgenic proteins, immunohistochemistry was performed using antibodies against p130 and E2F5. In the nontransgenic FVB lens, shown in the next section, there was no staining for p130 protein in fiber cells (Figure 6I), consistent with the previous observation [23]. By comparison, both p130 and E2F5 protein staining are present in lens fiber cell nuclei of the double transgenic mice (Figure 1F,G).

### Expression of E2F5/p130 decreases E2F3a-induced cell cycle reentry

Hypophosphorylated p130 forms a repressor complex with the E2F4, which can block E2F-responsive promoter sites, and can inhibit the G_1_/S transition of the cell cycle [18-20]. We hypothesized that the E2F5/p130 complex could also function as a cell cycle repressor in vivo. To examine whether coexpression of E2F5 and p130 can inhibit cell proliferation induced by E2F3a, E2F3a transgenic mice (family OVE1728) were cross-mated with the E2F5/p130 double transgenic mice (OVE1811) to generate triple transgenic mice. At E15.5, when compared to nontransgenic FVB lenses (Figure 7A,D), the E2F3a single transgenic lenses showed defects in fiber cell elongation and BrdU positive fiber cells (Figure 7B,E) [15]. The triple transgenic lenses showed less severe pathology (Figure 7C). Although condensed fiber cell nuclei were still present in the center of the triple transgenic lens (Figure 7C), the number of BrdU positive fiber nuclei was decreased by about 50% (p<0.01; Figure 7F; Table 1). Immunohistochemistry showed staining for Myc-tagged E2F3a, E2F5 and p130 proteins in most of the triple transgenic lens fiber cell nuclei (Figure 6C,G,K) when compared to the non-transgenic FVB lenses (Figure 6A,E,I), confirming the expression of these transgenic proteins.

### Expression of E2F5/p130 inhibits E2F1-induced cell cycle reentry

To test whether coexpression of E2F5 and p130 could also inhibit E2F1-induced cell cycle reentry, E2F1 transgenic mice (family OVE527) were cross-mated with the E2F5/p130 double transgenic mice from family OVE1811 to generate triple transgenic mice. At E15.5, the triple transgenic lenses still showed defects in fiber cell elongation and alignment, although slightly less severe than the E2F1 single transgenic lenses (Figure 8A,B) [29]. Condensed fiber cell nuclei were still present in the center of the triple transgenic lens (Figure 8B), indicating that these cells were undergoing programmed cell death. However, the number of BrdU positive fiber cell nuclei in the triple transgenic lenses was decreased by 80% (p<0.01; Figure 8C,D; Table 1) [29], suggesting that E2F5/p130 expression causes a dramatic reduction in E2F1 activity. The expression of E2F1, E2F5 and p130 transgenes in the triple transgenic lens fiber cells was confirmed by in situ hybridization (Figure 9). When compared to the intensity of E2F1 or E2F5 transcription signals (Figure 9B,D), the level of p130 transgene expression was low (Figure 9F).

## Discussion

E2F4 and E2F5 are thought to be cell cycle repressors as E2F4^−/−^ and/or E2F5^−/−^ cells are unable to exit the cell cycle in response to p16^INK4A^ [21]. Our previous transgenic studies demonstrated that E2F4 can function as a weak cell cycle activator and can induce cell cycle reentry in post-mitotic lens fiber cells [15]. In this study, we have expressed E2F5, with/without p130, together with E2F1 or E2F3a in the ocular lens of transgenic mice to assess the prediction that E2F5 can inhibit or repress cell cycle progression. Our results indicate that overexpression of E2F5 alone was not sufficient to inhibit cell cycle reentry induced by the cell cycle activators E2F1 and E2F3a. Bigenic expression of E2F5 and p130 in lens fiber cells did not affect normal fiber cell differentiation. However, overexpression of E2F5 and p130 provided 50 – 80% inhibition of E2F1 or E2F3a induced cell cycle entry. Our transgenic studies provide in vivo evidence that E2F5/p130 can function as a cell cycle repressor and can inhibit cell proliferation. Our studies do not address the issue of whether p130 alone can inhibit cell cycle reentry induced by E2F1–3, or whether this inhibition requires formation of a p130-E2F5 complex.

### E2F5 alone does not repress cell cycle progression

When the pRb family members are phosphorylated and dissociated from the E2Fs, the free E2F4 and E2F5 proteins move to the cytoplasm, while the activator E2F proteins (E2F1, E2F2, and E2F3a) bind to the E2F-responsive gene promoters that are vacated by E2F4 and E2F5, even though at some promoters their binding sites may be different [35,36]. We have shown that expression of E2F1–4 in ocular lens fiber cells can upregulate E2F target genes and induce post-mitotic lens fiber cells to re-enter the cell cycle [15,29]. In this study, we used the same transgenic approaches to test whether extra E2F5 could interfere with the activator E2Fs. We found that overexpression of E2F5 in fiber cells was not sufficient to disrupt S phase induction by E2F1 or E2F3a.

### Generation of E2F5/p130 double transgenic mice

Since extra “free” E2F5 did not interfere with the activator E2Fs, we hypothesized that the role of repressor E2Fs might be dependent upon their binding partners, the pocket proteins. Since E2F5 is believed to bind preferentially to p130 [8], we used the δenαA-minx promoter (DREAM) vector for E2F5 and p130 cDNA constructs to generate E2F5/p130 double transgenic mice. This modified αA-crystallin promoter has been shown to be active in both lens epithelial and fiber cells [30]. However, among transgenic mice (OVE1811 and OVE1812) that were obtained from our micro-coinjection, we found that both E2F5 and p130 transgenes were mainly expressed in lens fiber cells, with little or no expression in lens epithelial cells (Figure 1). Inactivation of DREAM promoter in lens epithelial cells might be due to different transgene integration site, or repressive interaction between E2F5 and p130 transgenes.

### E2F5/p130 does not affect normal lens fiber differentiation, but inhibits deregulated cell cycle progression

Expression of E2F4 and E2F5 is found mainly present in quiescent cells at G_0_, and is thought to be important for differentiation, because mice that are nullizygous for these E2Fs have specific terminal-differentiation defects [37,38]. The pRb homologs, p107 and p130, can form repressor complexes in conjunction with E2F4 or E2F5 at most if not all the E2F-responsive gene promoters [9]. Vairo et al. [18] showed that p130 can interact with E2F4, and can actively repress E2F-regulated promoter activities. In our transgenic study, we found that when E2F5 and p130 were overexpressed in post-mitotic lens fiber cells, there was no inhibition of fiber cell differentiation or elongation. However, coexpression of E2F5 and p130 did significantly repress the cell cycle progression induced by overexpression of E2F1 or E2F3a. These results indicate that the activity of cell cycle “repressor” E2F5, if any, is dependent upon the tumor suppressor p130. Overexpression of p130 was previously found to arrest cancer cells in the G_1_ phase of the cell cycle and inhibit tumor growth [2,16,17]. Our transgenic observations are consistent with these previous studies. However, so far it is unclear whether p130 can inhibit cell cycle progression in the absence of E2F5. It was reported that in the absence of both E2F4 and E2F5, hypophosphorylated pocket proteins were unable to induce cell cycle exit [21], suggesting that these repressor E2Fs may be a necessary mediator for the pocket protein function. The precise growth suppressive mechanisms of p130 are not fully understood. One possibility is that it forms a repressive complex with E2F4 and E2F5, which then recruits HDAC1 to the E2F-regulated gene promoter and repress its activities [39]. Alternatively, since E2F1, E2F2, and E2F3 are capable of binding to p130 in two-hybrid systems or when overexpressed [40,41], the other possibility is that p130 binds directly to E2F1 and E2F3a to eliminate their transactivation ability. Nevertheless, how p130 interplays with E2Fs to function as a tumor suppressor remains to be determined.

### E2F5/p130 may be a useful tool in anti-cancer gene therapy

Our in vivo transgenic results may have clinical significance. Mutations in the human gene encoding p130 are detected in diverse cancers including prostate, breast, and ovarian cancers [2]. Several studies have shown that p130 is downregulated in tumors at the transcriptional level rather than by posttranslational modifications [42-44], suggesting that loss of p130 expression could be an important event in development of tumors. For example, downregulation of p130 has been found in the genesis of neoplasms of the lung [43], breast [45], ovary [42], and non-Hodgkin’s lymphomas [46]. Development of mesothelioma [47] and AIDS-related lymphomas [46] is also related to inactivation of p130 function by viral oncoproteins. A previous study showed that retrovirus-mediated delivery of wild type p130 to the lung tumor cells could suppress tumor growth in vitro and in vivo [17], indicating a role of p130 in gene therapy. Our transgenic observations further confirm that p130, when overexpressed, can reduce inappropriate cell cycle reentry. Therefore, it may be a useful tool in gene therapy to inhibit tumor growth.

Overexpression of E2F1 or E2F3 has been shown to lead to both p53-dependent and – independent apoptosis in tissue culture cells [11,48]. E2F1 was found to increase transcription of p53 [49] and to induce p53 protein accumulation through activation of p19^ARF^ [11]. In addition, the p73 promoter region contains several E2F-binding sites, and overexpression of E2Fs can transactivate the p73 promoter [48]. In our previous transgenic studies, we showed that expression of E2F1 and E2F3a in post-mitotic lens fiber cells is sufficient to induce cell cycle reentry, followed by p53 and p73 stabilization and cell death [15,29]. In the current study, we did not do TUNEL assays to analyze changes in apoptosis. However, the many condensed nuclei in the triple transgenic lens fiber cells (Figure 7 and Figure 8) indicate that programmed cell death is still occurring. This suggests that overexpression of p130 may not interfere with E2F induced apoptosis, which would enhance the anti-tumor activity of p130.

In summary, we have used the transgenic mouse lens as a model system to study E2F and p130 activities. Although E2F5 is one of the “repressor” E2Fs, expression of E2F5 alone was not sufficient to interfere with cell cycle induction by the activator E2Fs. Overexpression of both E2F5 and p130 did not affect normal post-mitotic lens fiber cell differentiation. However, they did inhibit E2F-induced cell cycle reentry. Although the molecular basis of this phenomenon is not yet fully understood, our studies support the notion that p130 is an important cell cycle repressor, and therefore, may be useful for gene therapy in the treatment of tumors.
